# Totally endoscopic treatment of duodenal diverticulum

**DOI:** 10.1055/a-2155-4535

**Published:** 2023-09-15

**Authors:** Mario Capasso, Lorenzo Dioscoridi, Edoardo Forti, Francesco Pugliese, Marcello Cintolo, Giulia Bonato, Massimiliano Mutignani

**Affiliations:** Digestive and Interventional Endoscopy Unit, ASST Niguarda Hospital, Milan, Italy

Intraluminal duodenal diverticulum, also called “windsock” diverticulum, is an extremely rare congenital abnormality 1 . It is mostly asymptomatic but in 10 % of cases it can cause epigastric pain, upper gastrointestinal mechanical obstruction, and, less commonly, weight loss and biliopancreatic symptoms 2 . Surgery is considered the gold standard treatment for selected symptomatic cases 3 . Endoscopic excision is not standardized in the literature and only case reports have been published 4 .

A 39-year-old woman without relevant comorbidities reported pancreatitis-like symptoms with recurrent post-prandial epigastric pain associated with vomiting and weight loss (about 7 kg in 2 months). Blood tests showed a high level of lipase. She underwent endoscopic and radiological evaluation for suspected nonbiliary pancreatitis.

On esophagogastroduodenoscopy (EGD), a large diverticulum was identified beyond the upper duodenal knee, causing almost complete obstruction of the second portion of the duodenum and with a hole at the bottom probably caused by food inflow. Magnetic resonance cholangiopancreatography confirmed a duodenal diverticulum 7 cm in length ( Fig. 1 ) and revealed the major papilla immediately below the diverticulum. The major papilla was not identified during the endoscopic examination, and subsequent endoscopic retrograde cholangiopancreatography failed to provide further diagnostic information.

**Fig. 1:**
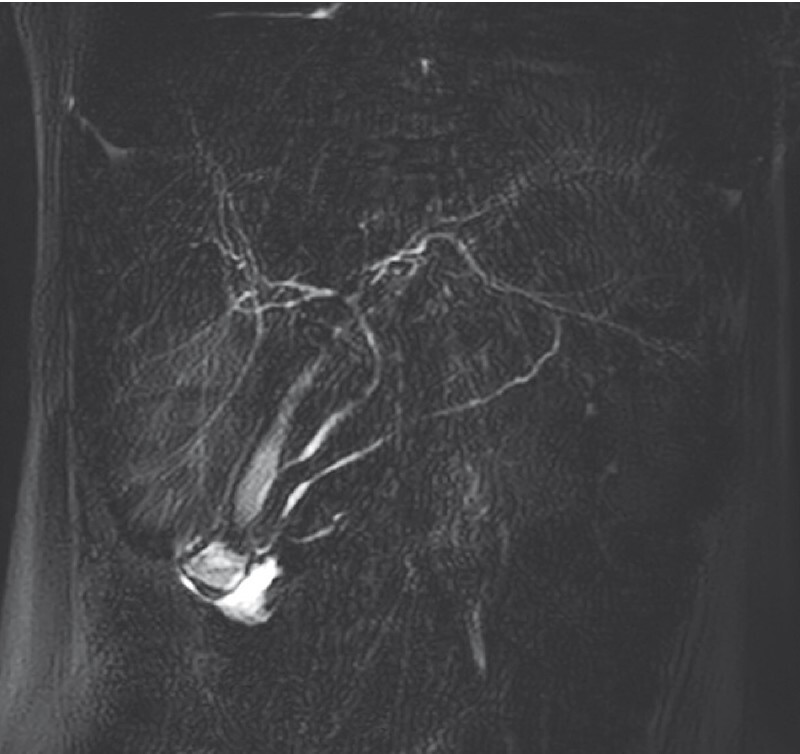
Magnetic resonance cholangiopancreatography showed a large duodenal diverticulum in the area of the papilla of Vater.

Surgery was proposed; however, given her recent malnutrition, the patient chose, following our center’s suggestion, endoscopic removal as the initial approach ( Video 1 ).

**Video 1** Large symptomatic duodenal diverticulum treated totally by endoscopic resection.


Prior to the procedure, a 15 mm Fogarty balloon was passed into a gastroscope with a 3.8 mm operative channel, and an open 30 mm endoloop was premounted into a duodenoscope with a 4.2 mm operative channel. The gastroscope was introduced and the Fogarty balloon was passed through the hole at the bottom of the diverticulum and inflated to turn the diverticulum inside out. The gastroscope was removed, leaving the Fogarty balloon in place. The duodenoscope was introduced and the endoloop was passed around the Fogarty balloon and applied at the base of the reverted diverticulum. Finally, the diverticulum was resected around and 1 cm beyond the closed endoloop using an insulated tip knife. Hemostatic prophylaxis of the resected edges was performed by placing 11 mm through-the-scope endoclips (Micro-Tech Co, Ltd, Nanjing, China) with flexible tips ( Fig. 2 ).

**Fig. 2:**
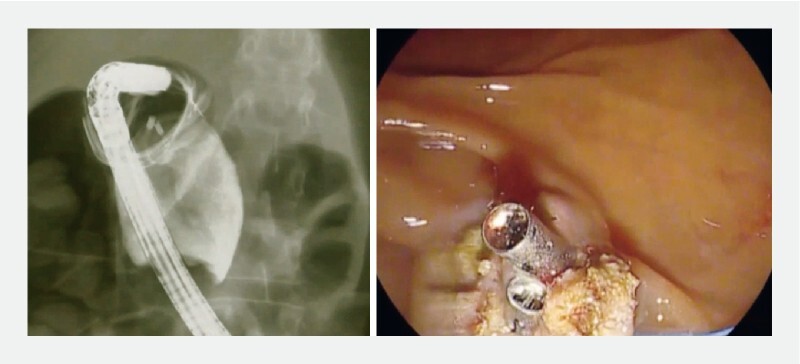
At the end of resection, through-the-scope endoclips were placed. No parietal perforation on contrast X-ray evaluation was observed.

On the first postoperative day, the patient experienced a severe episode of hematemesis. Repeat EGD showed diffuse bleeding from the edges due to partial loosening of the endoloop. Further endoclips were placed. The patient started eating on the second postoperative day and no further adverse events were reported. The patient was discharged on the third postoperative day.

At the 6-month follow-up, the patient was in good clinical condition without any of the presenting symptoms and had regained the lost weight. EGD showed a linear scar at the site of the diverticulum and a regular papilla major ( Fig. 3 ).

**Fig. 3:**
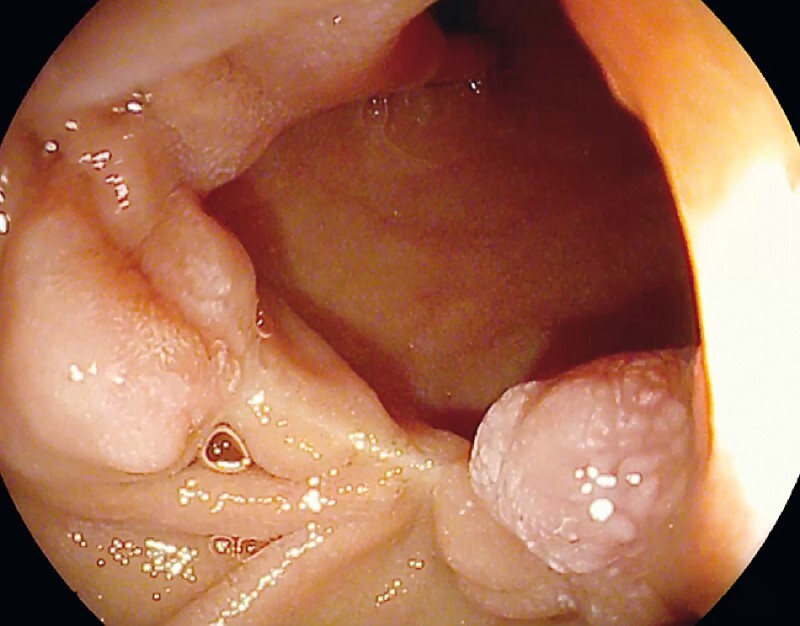
The 6-month follow-up esophagogastroduodenoscopy showed a mucosal scar at the resection site and regular papilla major. The patient had complete relief of symptoms.

Endoscopic resection of symptomatic windsock diverticula can be performed in tertiary referral endoscopy centers. According to the literature 5 , post-procedural bleeding is a common adverse event and can be managed endoscopically.

Endoscopy_UCTN_Code_TTT_1AO_2AG

## Notes

Endoscopy E-Videos

https://eref.thieme.de/e-videos
*E-Videos* is an open access online section of the journal *Endoscopy* , reporting on interesting cases and new techniques in gastroenterological endoscopy. All papers include a high-quality video and are published with a Creative Commons CC-BY license. Endoscopy E-Videos qualify for HINARI discounts and waivers and eligibility is automatically checked during the submission process. We grant 100% waivers to articles whose corresponding authors are based in Group A countries and 50% waivers to those who are based in Group B countries as classified by Research4Life (see: https://www.research4life.org/access/eligibility/ ). This section has its own submission website at https://mc.manuscriptcentral.com/e-videos
